# Random mutagenesis in *Corynebacterium glutamicum *ATCC 13032 using an IS*6100*-based transposon vector identified the last unknown gene in the histidine biosynthesis pathway

**DOI:** 10.1186/1471-2164-7-205

**Published:** 2006-08-10

**Authors:** Sascha Mormann, Alexander Lömker, Christian Rückert, Lars Gaigalat, Andreas Tauch, Alfred Pühler, Jörn Kalinowski

**Affiliations:** 1 Institut für Genomforschung, Universität Bielefeld, D-33594 Bielefeld, Germany; 2 Lehrstuhl für Genetik, Universität Bielefeld, D-33594 Bielefeld, Germany

## Abstract

**Background:**

*Corynebacterium glutamicum*, a Gram-positive bacterium of the class Actinobacteria, is an industrially relevant producer of amino acids. Several methods for the targeted genetic manipulation of this organism and rational strain improvement have been developed. An efficient transposon mutagenesis system for the completely sequenced type strain ATCC 13032 would significantly advance functional genome analysis in this bacterium.

**Results:**

A comprehensive transposon mutant library comprising 10,080 independent clones was constructed by electrotransformation of the restriction-deficient derivative of strain ATCC 13032, *C. glutamicum *RES167, with an IS*6100*-containing non-replicative plasmid. Transposon mutants had stable cointegrates between the transposon vector and the chromosome. Altogether 172 transposon integration sites have been determined by sequencing of the chromosomal inserts, revealing that each integration occurred at a different locus. Statistical target site analyses revealed an apparent absence of a target site preference. From the library, auxotrophic mutants were obtained with a frequency of 2.9%. By auxanography analyses nearly two thirds of the auxotrophs were further characterized, including mutants with single, double and alternative nutritional requirements. In most cases the nutritional requirement observed could be correlated to the annotation of the mutated gene involved in the biosynthesis of an amino acid, a nucleotide or a vitamin. One notable exception was a clone mutagenized by transposition into the gene *cg0910*, which exhibited an auxotrophy for histidine. The protein sequence deduced from *cg0910 *showed high sequence similarities to inositol-1(or 4)-monophosphatases (EC 3.1.3.25). Subsequent genetic deletion of *cg0910 *delivered the same histidine-auxotrophic phenotype. Genetic complementation of the mutants as well as supplementation by histidinol suggests that *cg0910 *encodes the hitherto unknown essential L-histidinol-phosphate phosphatase (EC 3.1.3.15) in *C. glutamicum*. The *cg0910 *gene, renamed *hisN*, and its encoded enzyme have putative orthologs in almost all Actinobacteria, including mycobacteria and streptomycetes.

**Conclusion:**

The absence of regional and sequence preferences of IS*6100*-transposition demonstrate that the established system is suitable for efficient genome-scale random mutagenesis in the sequenced type strain *C*.*glutamicum *ATCC 13032. The identification of the *hisN *gene encoding histidinol-phosphate phosphatase in *C. glutamicum *closed the last gap in histidine synthesis in the Actinobacteria. The system might be a valuable genetic tool also in other bacteria due to the broad host-spectrum of IS*6100*.

## Background

*Corynebacterium glutamicum *is a non-sporulating soil bacterium, belonging to the high G+C Gram-positive Actinobacteria. The bacterium, originally isolated as a natural glutamic acid excreter, is nowadays widely used as an industrial relevant producer of several L-amino acids and vitamins [1,2]. The common method to isolate amino acid-producing strains of *C. glutamicum *uses the iterative approach of general mutagenesis and subsequent screening for mutants with a high capacity to secrete the amino acid of interest [3]. With the development of genetic engineering methods, like gene disruption and replacement [4,5] or over-expression and deregulation of single genes [6,7], tools for more rational strain improvement are provided and successfully applied [8,9]. The availability of the complete genome sequence of *C. glutamicum *ATCC 13032 in combination with automatic annotation tools [10] enhanced the potential of corynebacterial research greatly. However, despite single gene manipulation techniques and the acquired knowledge about genes or the function and regulation of their corresponding enzymes in the metabolic context of the cell, there are still gaps in some metabolic pathways and a large amount of genes for which functions could not yet be assigned. A practicable strategy to analyse genes of unknown function is to mutate them, e.g. by generating a genomic insertion mutant library, and subsequently characterize the phenotypic properties of individual clones within the library. An ideal resource for this purpose would be an ordered or random collection of insertion mutants, each containing a defined insertion in a particular non-essential gene, with which a large number of clones could be analysed simultaneously [11,12].

In response to this demand, transposon mutagenesis systems became of increasing interest for genome-scale manipulation, analyses and single-gene studies in bacteria. Transposon systems are efficient mutagenesis tools in a wide variety of bacterial species [13]. The advantages of a mutagenesis system employing antibiotic resistance conferring transposons are the ability of a random integration into the chromosome, a positive selection for transposon mutants and the physical marker introduced at the site of mutation [14].

Transposons and insertion sequences (IS) are defined segments of DNA that can relocate as a unit between genomic regions [15]. Transposons range from class I composite transposons consisting of a pair of IS elements that enclose additional genetic information for antibiotic resistance or other properties, to complex class II transposons, to conjugative transposons that combine hybrid features of transposons, plasmids and bacteriophages [16].

Insertion sequences (IS) are the simplest form of mobile genetic elements. These elements generally possess one or two open reading frames encoding the protein that causes its mobility, the transposase. The majority of IS elements is flanked by terminal inverted-repeat sequences (IR). As a result of the integration mechanism IS generate directly repeated sequences (DR) of the target DNA flanking the element of a fixed length, which are characteristic for a given element.

A large number of native insertion sequences and their isoforms as part of transposons could be isolated from chromosomes and plasmids of coryneform bacteria and assigned to different members of mobile element families [17]. Furthermore, insertion sequences can be used to construct artificial transposons [18] and artificial transposon vectors [19] to circumvent, for instance, the natural limitation of missing antibiotic resistance markers. Several active mobile IS have been published for *C. glutamicum*. Most of these appear to transpose randomly, but are of limited usability for random mutagenesis due to a more or less distinctive target site preference. The IS elements IS*1249 *and IS*1513 *as part of the transposons Tn*5432 *and Tn*5564*, prefer triple A/T or a central palindromic tetranucleotide (CTAG) as target sequences [20,21]. Recent studies with IS*31831*, as part of Tn*14751 *or IS*14999 *revealed A/T-rich regions [22] or an 8-bp palindromic sequence as preferred target sites [23].

In 2003, Bonamy *et al*. [24] reported about an IS*1207*-based transposon Tn*5531 *with an apparent low target site-specificity in the *C. glutamicum *strain ATCC 14752. However, the endogenous occurrence of seven copies of the highly similar insertion sequences (IS*Cg1a *– IS*Cg1d*, IS*Cg7*, IS*Cg10 *and IS*Cg17*) in the strain ATCC 13032 impede the application of this transposon there since integrations would mainly be a result of RecA-mediated homologous recombination events.

The insertion element IS*6100 *was initially isolated as part of the composite transposon Tn*610 *of *Mycobacterium fortuitum *[25]. Later studies revealed its presence in a wide spectrum of host organisms from different bacterial lineages, e.g. *Arthrobacter *sp. [26],* Pseudomonas aeruginosa *[27], *Xanthomonas campestris *[28], or *Aeromonas salmonicida *[29]. It is 880-bp in size and is bordered by 14-bp perfect terminal inverted repeats. In common with other members of the IS*6 *family it translocates by replicative transposition which results in both the formation of a cointegration complex with an additional copy of the element itself as an end-product and the occurrence of an 8-bp direct repeat at the target site [17,30]. Tauch *et al*. [31] isolated IS*6100 *from the *C. glutamicum *antibiotic resistance-plasmid pTET3 present in the strain *C. glutamicum *LP-6 and showed that the element is transpositionally active in the strain ATCC 13032. Subsequent Southern hybridization analyses of a small number of clones suggested that IS*6100 *inserts in a random manner into the *C. glutamicum *chromosome.

In this study a transposon library of the completely sequenced type strain ATCC 13032 with a statistically representative size was constructed using an transposon vector based on the IS*6100 *element. By auxanography analyses several auxotrophic mutants could be obtained. Plasmid rescue and sequencing of the insertion sites revealed a number of known biosynthesis genes as well hitherto unknowns. The additional development of a PCR-based screening strategy permitted the rapid detection of insertion mutants in any chromosomal region of interest. Taken together, the transposon used and the library created represent novel tools with high impact on functional genome analyses in *C. glutamicum *ATCC 13032.

## Results

### Transposon mutagenesis of *Corynebacterium glutamicum* ATCC 13032 with an IS*6100*-based transposon vector

*In vivo *mutagenesis of the *C. glutamicum *ATCC 13032 genome was performed with the IS*6100-*based artificial transposon vector named pAT6100. The vector is a construct resulting from cloning IS*6100 *into the vector pK18*mob2 *[31]. The pAT6100 plasmid DNA extracted from *Escherichia coli *DH5αMCR was transformed into electrocompetent cells of *C. glutamicum *RES167, a restriction-deficient strain derived from the wild-type, by electroporation. Since the vector shares no homologous sequences with the host genome and it is not able to replicate autonomously in *C. glutamicum *the kanamycin resistance phenotype is the result of a transposition event into the chromosome. The transposition efficiencies ranged from five to ten c.f.u. per μg of desalted plasmid DNA.

An ordered clone library was constructed comprising 10,080 independent mutant clones. With a coverage more than three-fold with respect to the 3,002 coding regions determined for *C. glutamicum *ATCC 13032 [10] we expect that nearly all non-essential genes contain insertions. In order to estimate the theoretical quality of the library, statistical analyses were made: Given the number of clones in the transposon library (10,080), the estimated number of non-essential genes (2,400) [32], and the average gene length (952 bp), the average probability for any given gene to be disrupted by at least one transposon insertion is 97%.

**Figure 1:**
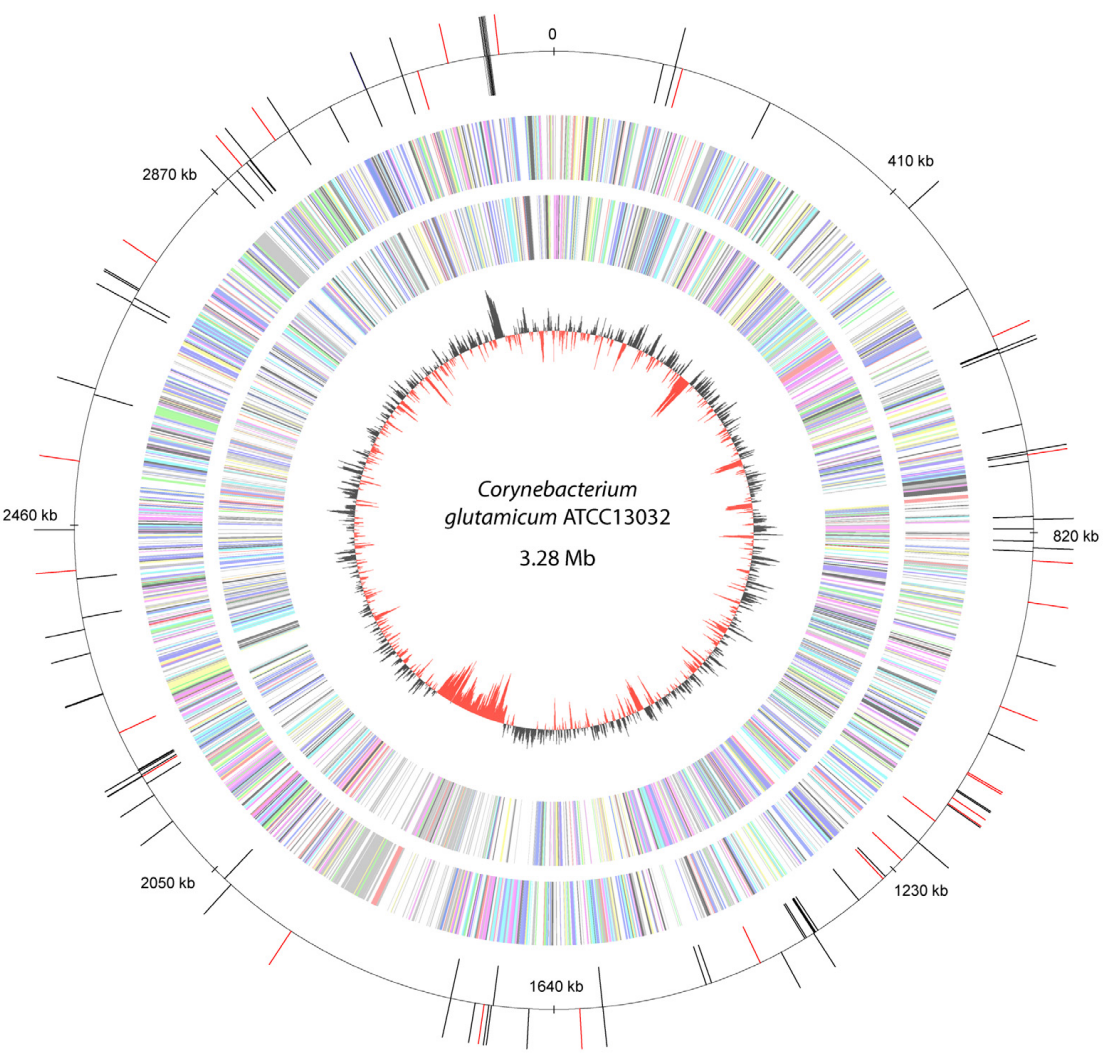
Distribution of transposon insertions on a circular plot of the *C. glutamicum *ATCC 13032 genome [GenBank:BX927147]. Coloured bars of the outer circle pointing inward and outward show the orientation of the cointegrates with respect to the genome in clockwise and counterclockwise direction, respectively. Red bars indicate the integration positions mapped for randomly selected clones and black bars map those investigated by auxotrophy analysis. Additional circles (from inward to outward) represent relative G+C content and coding regions transcribed in clockwise and counterclockwise direction, respectively. A positive deviation in G+C content from the average (53.8%) is shown by bars pointing outward and a negative deviation by bars pointing inward. Annotated genes are coloured according to the colour scheme of the functional classes system COG (cluster of orthologous groups) [89]. The plot was generated with GenDB version 2.2 [90].

In order to perform experimental analyses of the library, first a potential target site preference of the transposon was tested. Therefore, 26 clones were randomly selected and plasmid rescue cloning was carried out by digesting chromosomal DNA of the selected clones with EcoRI or XbaI, respectively, religating of the rescue constructs, transferring to *E. coli *and subsequent sequencing of the genomic insert with IS*6100*-specific primers. The sequence data obtained was analysed for sequence homologies with BLASTN searches against the *C. glutamicum *ATCC 13032 whole genome sequence [10]. The BLAST results revealed the common 14-bp inverted repeat of either left and right flank of the cointegrate followed by the individual 8-bp direct repeat target site for each clone, indicating the position of the transposon integration (Table 1). As the target sites of the clones were each different, with no duplicates with the same or similar sequences, it can be assumed that these 26 transposition events occurred independently. The position of the transposon in every mutant, as well those selected randomly (Fig. 1, red bars) and those investigated in auxanographic analyses (Fig. 1, black bars) were plotted on a circular map representing the *C. glutamicum *ATCC 13032 chromosome. The picture indicates a random distribution throughout the genome without regions of significant accumulation of insertions. Both, intragenic and intergenic insertions were detected. The G+C content was calculated from the eight nucleotides of the target site and ten flanking nucleotides upstream and downstream. It showed a high variance ranging from 39.3% to 78.5%, whereas the medium G+C content for the chromosome of the strain ATCC 13032 is 53.8%. The two possible orientations of the cointegrate with respect to the directions of replication or transcription are present in nearly equal proportions indicating that neither orientation is favoured.

**Table 1:** Target site analyses of randomly selected *C. glutamicum *transposon mutants.

Clone notation ^a^	Integration position ^b^	Locus tag ^c^	Gene	Gene product	Target site duplication (TSD) ^d^	G+C content [%] ^e^	Transposon orientation ^f^
51G06	141,831	*cg0165*		ABC-2 type transporter	GGCGCGCG	78.5	-
01A01	603,250	*cg0683*		Permease	AGTGAACC	53.5	+
52D11	737,930	*cg0822*		Conserved hypothetical protein	CCCAACGG	60.7	+
51B11	853,795	*cg0924*		ABC-type cobalamin/Fe^3+^-siderophores transport system periplasmic components	CTCAACGG	53.6	+
31D11	899,182	*cg0963_0964*			CTCTTTTT	39.3	+
51H01	1,097,334	*cg1185*	*tnp10b*	Transposase – fragment IS*Cg10a*	AAAAATAC	57.1	+
51D02	1,099,353	*cg1192*		Aldo/keto reductase	CCCTAGCG	46.5	+
147A01	1,129,669	*cg1228*		ABC-type cobalt transport system, ATPase component	CCTACGTT	42.9	+
51B03	1,137,471	*cg1237*		Putative membrane protein	CCTTTGAG	42.9	+
51E04	1,161,219	*cg1265*		Conserved hypothetical protein	TAAGGAAG	39.2	-
51E02	1,217,257	*cg1310*	*tfdF*	Maleylacetate reductase	CGATTACG	50.0	-
51G12	1,246,055	*cg1338*	*thrB*	Homoserine Kinase	CCTATTAC	46.4	-
57D08	1,408,925	*cg1516*		Hypothetical protein	GCGATATC	46.4	-
121D05	1,613,084	*cg1724*		Putative protein kinase ArgK or related GTPase of G3E family	AGGTGAAG	60.7	+
43C10	1,717,632	*cg1825*	*efp*	Translation elongation factor P	CGGGTGTC	60.7	+
157H05	1,944,316	*cg2053_2054*		upstream putative membrane protein (*cg2054*)	CTTGATTC	42.9	+
29F07	2,180,143	*cg2296*	*hisI*	Probable phosphoribosyl-AMP cyclohydrolase	CGCCAAAG	57.1	-
17B06	2,234,105	*cg2349*		ATPase components of ABC transporters with duplicated ATPase domains	TTCTGGAA	64.3	-
75E09	2,418,388	*cg2537*	*brnQ*	Branched-chain amino acid uptake carrier	GTTTCATT	42.8	+
51C02	2,537,800	*cg2661*		Putative dithiol-disulfide isomerase involved in polyketide biosynthesis	ACTGGACT	53.6	+
51A01	2,772,437	*cg2913*		ABC-type Mn^2+^/Zn^2+ ^transport system, permease component	TTGCTGAT	57.2	+
97B06	2,913,169	*cg3049_3050*		upstream acyltransferase (*cg3050*)	CTCGACTG	57.1	+
02A09	2,958,982	*cg3100*	*dnaK*	Heat shock protein Hsp70	TTCTCAGC	53.5	+
51A02	3,132,229	*cg3271_3272*			GTCAGCCG	60.7	-
35E03	3,166,513	*cg3319_3320*		upstream uncharacterized enzyme related to sulfurtransferases (*cg3319*)	CTTCAGTC	46.4	+
51F01	3,222,657	*cg3373*		Bacterial regulatory protein, ArsR family	CTTCCGCG	60.8	+

^a ^Indicates the position of the clone in the transposon library.

^b ^Absolute position of the cointegrate in the *C. glutamicum *ATCC 13032 genome [GenBank:BX927147].

^c ^Integration of a transposon within or between genes (separated by underscoring)

^d ^Sequence of the 8-bp direct repeat.

^e ^G+C content was calculated from 28 nucleotides around integration (TSD and 10 nt down-/upstream).

^f ^Orientation of the integrated transposon with respect to the genome in clockwise (-) and counterclockwise (+) direction

An additional test for target site preferences was carried out by performing sequence pattern searches with the TEIRESIAS algorithm [33]. For this purpose a total of 172 target sites acquired from the random selected clones and from mutants of the auxanographic analyses (see below) were used. No sequence pattern, palindromic sequences or regions of nucleotide symmetry could be detected with this bioinformatic approach (data not shown). Additionally, the occurrence of the bases at a distinct position of the 8-bp target sequence was calculated (data not shown). The values and therefore the appearance of bases appear to be distributed evenly. Neither the pattern searches nor the nucleotide distributions in the TSD indicate a significant target site preference for insertion.

The cointegration of the transposon vector forms long direct repeats in the form of IS*6100 *copies at the site of insertion. Such direct repeats might cause instability by replication slippage or by homologous recombination, thereby losing the integrated vector and one or both of the IS*6100 *elements in the absence of antibiotic selection. To assess this, five randomly selected individual clones were subcultured in antibiotic-free liquid complex medium in twelve rounds of 48 h each. The last culture was plated on solid complex medium. From this culture, 900 single colonies from each clone were tested on kanamycin-containing plates. No loss of the resistance phenotype and therefore the integrated transposon could be observed. It has to be concluded that the integrations are stably maintained in *C. glutamicum *even in the absence of selective pressure. Additionally, isolated DNA from randomly selected clones which had not been grown under selective growth conditions was used to perform PCR experiments. In all cases the presence of the cointegrate was verified (data not shown).

### Auxanographic screening and targeted gene identification using PCR techniques

The initial step of performing genome-scale auxanographic analyses was the plating of the entire transposon library on minimal medium plates. After 32 h of incubation this screening approach delivered 342 mutants which exhibit a clear growth-deficient phenotype. The appearance of potential auxotrophic mutants which are unable to form colonies under such growth conditions was used as an initial criterion for efficient mutagenesis. These mutants were further characterized by the rapid method for identification of bacterial biochemical requirements according to Holliday [34]. Therefore, 12 plates of minimal medium were each supplemented with different combinations of six growth factors. Mutants with a single biochemical requirement would grow on two plates. The double auxotrophic mutants could be determined by growth on only a single plate and an alternative requirement by growth on more than two plates. For the latter two cases the exact nutritional requirements have to be determined by further tests combining individual growth factors.

With this method, a total of 36 different growth factors, which are adapted to corynebacterial needs by omitting biotin, choline, and thiosulfate, and adding cobalamine, glutamine, as well as asparagine, could be tested simultaneously. These growth factors comprise 21 amino acids, six nucleotides and nine vitamins, including some of their precursors. Each candidate auxotrophic clone was inoculated on all twelve plates and incubated for 32 h. Forty-seven out of the 342 clones tested formed slow growing colonies on every plate, suggesting that these are generally growth-deficient but not auxotrophic mutants. With the redefined number of 295 clones circa 2.9% of the transposon library are genuine auxotrophic mutants. This roughly correlates to prior studies with different transposons in which auxotrophic mutants were obtained with frequencies of 1.3% in *C. glutamicum *[24], 2.5% in *Rhodococcus spp*. [35] and 2% in *Rhodococcus fascians *[36].

Out of these 295 clones 167 display a requirement for a single growth factor (57%). Among these, 141 exhibit an auxotrophy for one of eleven different amino acids, 24 for one of four different nucleotides and two for different vitamins. Additionally, we have found twelve clones that show a double and six with an alternative requirement. Altogether, the auxotrophic requirements of nearly two thirds of the mutants could be identified.

From all of the auxotrophy categories a number of clones was selected and the transposon integration sites were determined using the plasmid rescue technique described above. Table 2 summarizes the phenotypic and genotypic data obtained from these clones.

**Table 2:** Characterization of transposon mutants with an auxotrophic phenotype

Number of clones	Auxotrophic requirement	Integrated in locus ^a^	Integrated in gene ^a^	Encoded protein	Functional complex	Reference
						
**Single auxotrophy**						
8	Adenine	*cg3063 cg2876*	*purA *(5) *purB *(3)	Adenylosuccinate synthase Adenylosuccinate lyase	AMP biosynthesis	[38]^e^
6	Guanine	*cg0703 cg0699 cg1216*	*guaA *(2) *guaB2 *(2) *nadA *(1)	putative GMP synthase, Inositol-monophosphate dehydrogenase, Quinolate synthase	GMP biosynthesis Quinolinate biosynthesis	[37]^d ^[91]^e^
8	Uracil	*cg1816 cg0617 *(1)	*pyrB *(1) *-*	Aspartate carbamoyltransferase catalytic chain, Putative Molybdopterin-guanine dinucleotide biosynthesis protein	Pyridmidine biosynthesis	[92]^e^
2	Hypoxanthine	*cg2857 *^b^	*purF *(1) ^b^	Amidophosphoribosyltransferase	Purine biosynthesis	[93]^f^
5	Arginine	*cg1586*	*argG *(1)	Argininosuccinate synthase	Arginine biosynthesis	[94]
6	Phenylalanine	*cg2391 cg3207*	*aroG *(2) *pheA *(4)	Phospho-2-dehydro-3deoxyheptonate aldolase, Prephenate dehydratase	Phenylalanine biosynthesis	[95] [96]
16	Histidine	*cg2299*, *cg2303*, *cg2305*, *cg1699*, *cg2297*, *cg1698*, *cg2296, cg0910*	*hisA *(1)*, hisB *(4),*hisD *(3)*, hisE *(1)*, hisF *(2)*, hisG *(2)*, hisI *(1), *hisN *(1)	Phosphoribosylformimin-5-aminoimidazole carboxamide ribotideisomerase, Imidazoleglycerol-phosphate dehydratase, Histidinol dehydrogenase, Anthranilate synthase component I, probabale Cyclase (imidazole glycerol phosphate synthase-subunit), Phosphoribosyltransferase, prob. Phosphoribosyl-AMP cyclohydrolase, Histidinol-phosphate phosphatase	Histidine biosynthesis	[97] [49]
		*cg2237*	*thiO *(1)	Putative D-amino acid oxidase flavoprotein oxidoreductase	Thiamine biosynthesis	[42]^g^
11	Proline	*cg0490*	*proC *(1)	Pyrroline-5-carboxylate reductase	Proline biosynthesis	[98]
5	Methionine	nd ^c^	nd ^c^			
13	Serine	*cg1451*	*serA *(1)	Phosphoglycerate dehydrogenase	Serine biosynthesis	[99]
17	Cysteine	*cg3118*	*cysI *(2)	Ferredoxin-sulfite reductase	Assimilatory sulfate reduction	[65]
14	Leucine	*cg1488*	*leuD *(1)	3-Isopropylmalate dehydratase, small chain	Leucine biosynthesis	[100]
6	Threonine	*cg1338*	*thrB *(1)	Homoserine kinase	Threonine biosynthesis	[101]
2	Lysine	*cg1334*	*lysA *(2)	Diaminopimelate decarboxylase	Lysine biosynthesis	[102]
46	Tryptophan	*cg3364*, *cg3363*, *cg3362*, *cg3361*, *cg3359*, *cg3360*	*trpA *(5)*, trpB *(11)*, trpCF *(10)*, trpD *(9),*trpE *(6), *trpG *(2)	Tryptophan synthase alpha chain, Tryptophan synthase beta chain, Indole-3-glycerol-phosphate synthase/phosphoribosylanthranilate isomerase, Anthranilate phosphoribosyltransferase, Anthranilate synthase component I, Anthranilate synthase component II	Tryptophan biosynthesis	[43]
1	Biotin ^h^	*cg2147 *(1)		BioY family membrane protein	Biotin transporter	
1	Pyridoxine	*cg0897*	*pdxR *(1)	Pyridoxine biosynthesis transcriptional regulator, Aminotransferase	Pyridoxine/valine biosynthesis	[47]
						
**Double auxotrophy**						
8	Arginine/Uracil	*cg1813 cg1814*	*carB *(1) *carA *(1)	Carbamoyl phosphate synthase subunit, Carbamoyl phosphate synthase small subunit	Arginine/pyrimidine biosynthesis	[103]^e^
4	Valine/Isoleucine	*cg1435 cg1437*	*ilvB *(2) *ilvC *(2)	Acetolactate synthase, Acetohydroxy acid isomeroreductase	Isoleucine/valine biosynthesis	[40]
						
**Alternative requirement**						
1	Arginine or Cytosine	nd ^c^				
1	Cysteine or Pyridoxine	*cg0156 *(1)		ROK-type transcriptional regulator	Cysteine/pyridoxine biosynthesis	
1	Proline or Threonine	*cg1238 *(1)		putative membrane protein		
3	Methionine or Cobalamin	*cg1290*	*metE *(2)	Homocysteine methyltransferase	Methionine biosynthesis	[41]

^a ^Numbers in brackets indicate the number of clones allocated to the respective gene.

^b ^Integration 30 nt upstream of start codon

^c ^nd, not determined.

^d ^Referenced for *Corynebacterium ammoniagenes*.

^e ^Referenced for *Escherichia coli*.

^f ^Referenced for *Mycobacterium smegmatis*

^g ^Referenced for *Rhizobium etli*.

^h ^Supplementable with 10-fold amounts of biotin compared to the wild-type strain

In the majority of clones the observed auxotrophic phenotype is well explained by the existing annotation of the disrupted gene. For example amino acid biosynthesis genes like *argG *(arginine), *thrB *(threonine), *proC *(proline), *serA *(serine), *leuD *(leucine) or *lysA *(lysine) were subject to intensive studies in *C. glutamicum *before, and the transposon insertions in these genes resulted in the expected auxotrophic phenotypes. This was also the case for insertions into genes that show high similarities to genes with a known function in closely related species, e.g. *guaA *and *guaB2*, known to be involved in the guanosine monophosphate biosynthesis in *Corynebacterium ammoniagenes *[37], or to genes in more remote organisms, e.g. *purA *and *purB *which are involved in adenosine monophosphate biosynthesis in *E. coli *[38]. Another example are the *carAB *genes: even though these genes are not characterized in *C. glutamicum *they are well known to cause a double requirement for both arginine and uracil in *E. coli *since they encode for the subunits of a protein that provides the essential carbamoylphosphate for arginine and pyrimidine biosynthesis [39]. The *ilvC *gene is well studied in *C. glutamicum *and is known to cause a double auxotrophy for isoleucine and valine when disrupted [40]. The successful supplementation of a *metE *transposon mutant strain with either methionine or cobalamin (vitamin B12) is explained by the fact that a cobalamin-dependent (MetH) and a cobalamin-independent enzyme (MetE) perform the same step in methionine biosynthesis in *C. glutamicum *[41]. The loss of the MetE enzyme, the preferred route during aerobic growth, can be compensated by supporting the MetH function with appropriate amounts of cobalamin.

In some cases the correlation of observed phenotype and the gene targeted by transposon integration are not so obvious. An example is the guanine auxotrophy of the *nadA *insertion. The *nadA *gene is located together with *nadB *and *nadC *and encodes a protein which is apparently involved in quinolinate and nicotinamide adenine dinucleotide biosynthesis. It is currently unclear why guanine supplements growth of this mutant. Also in the case of the integration in gene *thiO *the connection between gene function and auxotrophy is not clear yet. The gene as part of the *thiEOSGF *cluster encodes a putative D-amino acid oxidase flavoprotein oxidoreductase that is known to be involved in thiamine biosynthesis [42]. Although a polar effect on the downstream genes *thiSGF *is not experimentally verified, such an effect would furthermore affect a more extensive part of the thiamine synthesis. The mutant is supplementable by histidine and not, as expected, by thiamine.

Since the *de novo *purine and pyrimidine nucleotide synthesis pathways are poorly characterized in *C. glutamicum *especially the functions of genes like *purF *or *pyrB *require further studies as well. Mutants with transposon insertions in the *C. glutamicum *genes *purC*, *purE*, and *purK*, known as purine biosynthesis genes in *E. coli *[38], were found among those which grew on more than three plates (data not shown). These mutants appear interesting to be investigated in further studies.

**Figure 2:**
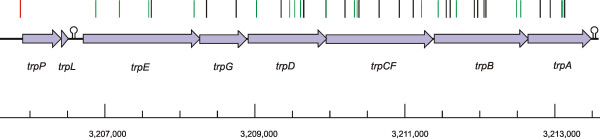
Physical map of the tryptophan gene cluster in *C. glutamicum *ATCC 13032. Within the *trpPEGDCFBA *genes, indicated by blue arrows, the coloured bars denote the mapped integrations of 43 independent tryptophan auxotrophic mutants. The two orientations with respect to the genome are indicated in black (clockwise) and green (counterclockwise direction). The transposon integration identified by PCR screening is also marked (orange bar). The left stem-loop symbol denotes the transcriptional attenuator involved in the regulation of the tryptophan biosynthesis [43] and the right loop a Rho-independent transcriptional terminator structure.

The genes of most biosynthetic pathways in *C. glutamicum *are scattered in different genetic loci. A special case where the distribution of transposon insertions in a limited genomic region could be tested is the *trp *operon containing six genes which encode all functions of tryptophan biosynthesis in *C. glutamicum *[43]. For this, the transposon insertion sites of 43 tryptophan-auxotrophic clones were determined. The insertion sites were all different and located within every gene from *trpE *to *trpA *(Fig. 2). Although the *trp *operon contains more insertions than statistically expected, this example gives additional evidence that the transposon library is random and that the transposon system can be used to mutagenise the *C. glutamicum *chromosome up to a high density.

To find a gene of interest that is mutated by a transposon is sometimes not possible by phenotypic screening. We were interested to map additional insertions upstream from the *trp *operon. For this, a PCR-based screening strategy was chosen and the workflow by Hobom *et al*. [44] was adapted so that the entire library could be screened for insertion events by iterative rounds of three-step PCR experiments simultaneously. This strategy delivered another transposon insertion which was mapped 35 bp upstream from the predicted *trpP *translation start. This mutant did not exhibit a tryptophan-auxotrophic phenotype in subsequent growth tests which coincides with the assumption that the *trpP *gene encodes a permease necessary for tryptophan- and 5-methyltryptophan uptake in *C. glutamicum *[45].

### Identification of a novel *C. glutamicum *gene closing the last gap in histidine biosynthesis

The whole genome sequence of *C. glutamicum *ATCC 13032 is known and the genes are annotated based on sequence similarity and experimental data [10]. Due to the fact that a large number of amino acid biosynthesis genes have been characterized before, only few pathways remained incomplete. With the help of sequence similarities and subsequent genetic analyses further genes have been identified [46] leaving only few gaps. These "missing" genes mostly encode transaminases which are notorious for their involvement in more then one pathway and were also partly characterized by bioinformatic and biochemical studies [47,48]. Beside this, only a single step in histidine biosynthesis remained, where no gene was known or a candidate gene could be identified by sequence similarity.

The metabolic pathway of the histidine biosynthesis, reactions, enzymes, as well as the organization of the corresponding genes display a high degree of conservation in bacteria [49]. According to the KEGG PATHWAY database [50] the histidinol-phosphate phosphatase (HolPase) is an exception which was not characterized at a genetic level in *C. glutamicum *and closely related Actinobacteria. This enzyme (L-histidinol-phosphate phosphohydrolase; EC 3.1.3.15) catalyses the penultimate step of the biosynthesis, the dephosphorylation of histidinol phosphate to histidinol, the direct precursor of histidine. In the reference organisms *E. coli *and *Salmonella typhimurium *the HolPase activity is associated with the N-terminal domain of the HisB bifunctional enzyme. The C-terminal domain exhibit similarity to an imidazoleglycerol-phosphate dehydratase (EC 4.2.1.19), which catalyses the sixth step of the histidine synthesis. However, amino acid sequence homology searches revealed that in *C. glutamicum*, like in most bacteria, the imidazoleglycerol-phosphate dehydratase is encoded as a monofunctional enzyme. Additional similarity searches for the *E. coli *homologs of the HolPase in *C. glutamicum *and other organisms, including the entire class of Actinobacteria, does neither provide any significant sequence homologies to ORFs in the *C. glutamicum *genome nor to well-conserved domains in other Actinobacteria.

**Figure 3:**
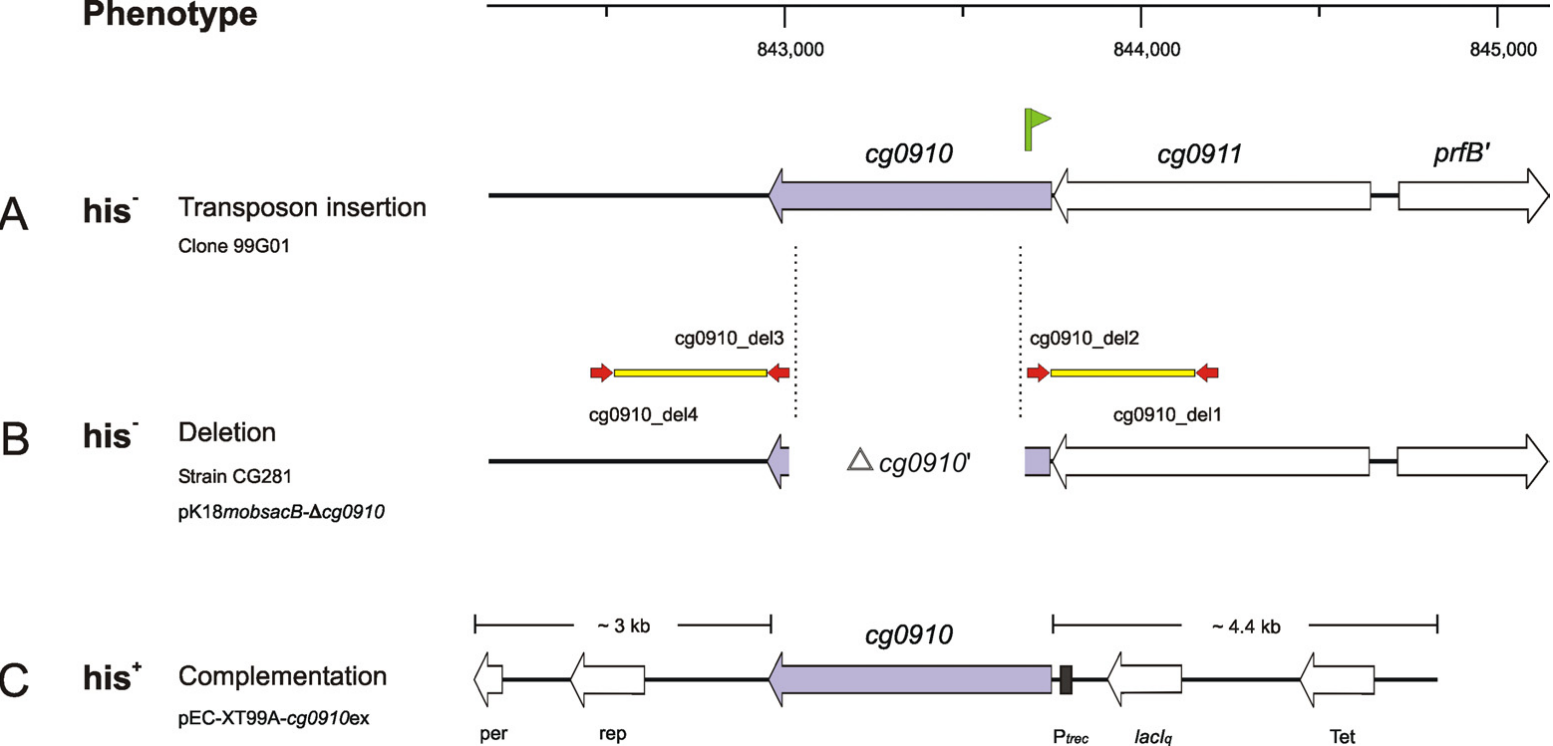
Schematic representation of the 3-kb chromosomal region around *cg0910 *(blue arrow) in *C. glutamicum*. His^- ^and his^+ ^denote a histidine-auxotrophic or -prototrophic phenotype of the corresponding mutant or strain, respectively. The transposon integration (A) is marked with a green flag. Red arrows indicate the binding positions of the primers used to amplify the deletion fragments (B; yellow boxes). The features of the deletion- (B) and complementation (C) constructs are also designated. The ruler indicates the absolute position in the genome.

Since the loss of the HolPase activity is supposed to exhibit a histidine-auxotrophic phenotype the corresponding unidentified gene in *C. glutamicum *was presumed to be among the 16 histidine-auxotrophic transposon mutants. Determination of the transposon integration sites in these mutants revealed insertions in seven of the nine histidine synthesis genes known so far, *hisGE*, *hisHAFI *and *hisDCB*, and another one in the gene *cg0910 *(Table 2). The integration in this gene, which comprises 783 bp, is located 125 bp downstream from the predicted *cg0910 *translation start, suggesting that the gene product is non-functional. The *cg0910 *gene is preceded by the gene *cg0911 *and both might be transcribed together (Fig. 3). The *cg0911cg0910 *locus is located far from known histidine biosynthesis genes. BLASTP searches with the deduced protein sequence of both genes revealed significant similarities to each other and to inositol-1(or4)-monophosphatases (IMP; EC 3.1.3.25) which hydrolyse the ester bond of inositol phosphate to generate inositol.

### Validation of *cg0910 *gene function by deletion and homologous complementation

In order to confirm the phenotype of the transposon mutation, a *cg0910 *deletion mutant was constructed using the "gene splicing by overlap extension" (gene SOEing) technique [51]. Therefore, a fusion product of chromosomal DNA regions of 622 bp and 541 bp directly up- and downstream, respectively, of the *cg0910 *target locus was created, which was subsequently cloned into the pK18*mobsacB *vector system [5]. The resulting vector pK18*mobsacB*-Δ*cg0910 *was finally used for targeted gene deletion. The deleted internal *cg0910 *fragment was 702 bp of size (Fig. 3).

The initially observed phenotype of the transposon mutant with the integration in *cg0910 *(clone 99G01) could be reproduced with the resulting deletion mutant, termed CG281, which was tested negative for its ability to grow on minimal medium without and positive with histidine supplementation. This indicated that possible secondary effects in the transposon mutant could be ruled out and that the loss of the *cg0910 *gene product is responsible for histidine auxotrophy. Both the mutants 99G01 and CG281 also grew on minimal medium supplemented with histidinol, the end product of the histidinol phosphatase reaction, comparable to the wild-type strain. These results suggest that *cg0910*, assigned with the gene name *hisN*, encodes the hitherto unknown but essential HolPase activity in *C. glutamicum*, and is the functional homolog of the *E. coli *N-terminal HisB protein domain (Table 2).

To provide further evidence that the *cg0910 *gene encodes the HisN function, homologous complementation was carried out in both mutants 99G01 and CG281. For this, the complete *cg0910 *coding sequence was amplified by PCR with primers providing an optimized ribosome binding site and additional restriction enzyme recognition sites. The amplificate was subsequently cloned into the IPTG-inducible shuttle expression vector pEC-XT99A [52]. The resulting plasmid pEC-XT99A-*cg0910*ex (Fig. 3) was subsequently transferred to the corresponding mutant strains which were then tested for histidine prototrophy. In both cases the extrachromosomally located *cg0910 *gene product complemented the loss of the corresponding chromosomal region *in trans *(data not shown).

### Phylogenetic analysis of Cg0910 and related inositol monophosphatases (IMP)

**Figure 4:**
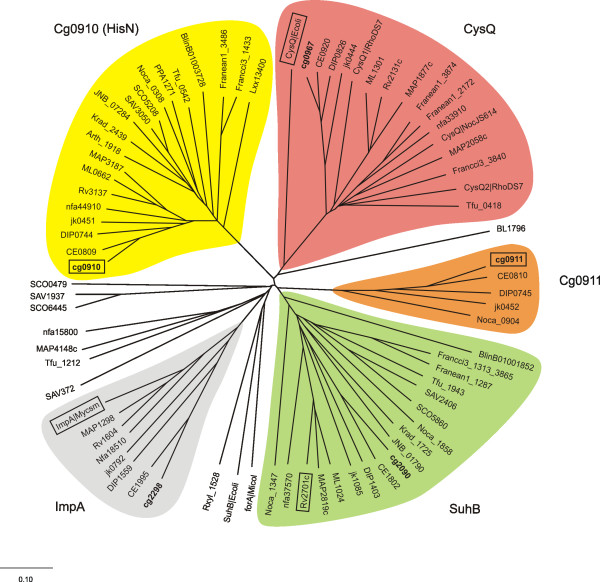
Dendrogram showing the relationship of inositol monophosphatase family proteins (IMP) in Actinobacteria and *E. coli *K-12. A multiple alignment with amino acid sequences of proteins with high similarity to the *C. glutamicum *IMPs was generated with the use of the DIALIGN2 software. Based on this alignment an unrooted phylogenetic tree was constructed using the neighbour-joining algorithm integrated in the CLUSTALX package and visualized as a radial tree by the TreeTool software. The branches were combined to classes that delivered within the bootstrapping analyses in at least two thirds of the cases the same subtree. These classes, marked by different colours, were named according to the designations in the boxed leaves. The locus tags (leaves) were obtained from the GenBank genome entries. *C. glutamicum *proteins are printed in bold letters. Locus tag prefixes denote following organisms (c, complete genome sequence; da, draft assembly): Arth (*Arthrobacter sp*. FB24; da), BL (*Bifidobacterium longum *NCC2705; c), BLinB01 (*Brevibacterium linens *BL2; da), DIP (*Corynebacterium diphtheriae *NCTC13129; c), CE (*C. efficiens *YS-314; c), cg (*C. glutamicum *ATCC 13032; c), jk (*C.  jeikeium *K411; c), Ecoli (*Escherichia coli *K-12; c), Francci3 (*Frankia sp*. CcI3; c), Franean1 (*Frankia sp*. EAN1pec; da), JNB (*Janibacter sp*. HTCC2649; da), Krad (*Kineococcus radiotolerans *SRS30216; da), Lxx (*Leifsonia xyli subsp. xyli *str. CTCB07; c), Micol (*Micromonospora olivasterospora*; da), MAP (*Mycobacterium avium subsp. paratuberculosis *K-10; c), ML (*M. leprae *TN; c), Rv (*M. tuberculosis *H37Rv; c), Mycsm (*Mycobacterium smegmatis str*. MC2 155; da), nfa (*Nocardia farcinica *IFM 10152; c), Noca (*Nocardioides sp*. JS614; da), PPA (*Propionibacterium acnes *KPA171202; c), RhoDS7 (*Rhodococcus sp*. DS7; da), Rxyl (*Rubrobacter xylanophilus *DSM 9941; da), SAV (*Streptomyces avermitilis *MA-4680; c), SCO (*S. coelicolor *A3(2); c) and Tfu (*Thermobifida fusca *YX; c).

BLAST searches revealed four IMP paralogs in the *C. glutamicum *ATCC 13032 genome with significant similarity to *cg0910*, by name *cg0911*, *cg0967 *(*cysQ*), *cg2090 *(*suhB*), and *cg2298 *(*impA*). For a functional classification of the inositol monophosphatase family-like proteins, phylogenetic analysis in comparison to sequence-related proteins from other Actinobacteria and the model organism *E. coli *was conducted. For this, similarity searches with the deduced amino acid sequences of the *C. glutamicum *IMPs in non-redundant databases were carried out, comprising, beside *E. coli *K-12, the genomes of Actinobacteria including completed as well as draft status genomes. Proteins with reliable similarity scores were used to perform a multiple alignment. Based on this alignment a phylogenetic tree was created by the neighbour-joining method and, subsequently, the tree was evaluated by bootstrap analysis (Fig. 4).

The phylogenetic tree separates the five *C. glutamicum *IMP paralogs (Fig. 4, bold) into distinct classes which all include apparent orthologs from other Actinobacteria. The denotation of the respective class was derived from members for which a physical function has been described (Fig. 4, box): CysQ in *E. coli *[53], ImpA in *Mycobacterium smegmatis *[54], SuhB in *M. tuberculosis *[55] and HisN (Cg0910) in *C. glutamicum*.

The putative orthologs of Cg0911 form a separate class. None of its members, comprising only corynebacterial and one *Nocardioides *species, was functionally characterized up to now.

## Discussion

In this study we describe a transposon system applicable for efficient random mutagenesis in *C. glutamicum *ATCC 13032. The use of an IS*6100*-based transposon vector gave rise to a transposon library of statistically representative size comprising independent mutant clones.

It was shown earlier that IS*6100 *is capable of transposing *in vivo *in *C. glutamicum *with unique transposition events by forming a cointegrate with the chromosome [31]. The excision frequency and therefore the stability of a replicative transposon integration is usually controlled by a resolvase protein, which is not encoded by the pAT6100 transposon vector. Nevertheless, cointegrate resolution involving either the two identical IS*6100 *copies or the 8-bp direct repeats might be caused by homologous recombination or by replication slippage resulting in the loss of the vector part and one or both copies of the IS*6100 *element. In this study an antibiotic sensitivity could not be observed after prolonged growth in the absence of antibiotic pressure during cultivation, indicating that IS*6100 *generates cointegrations that are stably maintained in *C. glutamicum*. This finding conforms with the study of Weaden and Dyson [56] in which the stable cointegration of IS*6100 *was observed in *Streptomyces avermitilis*.

The already developed transposon systems are not well applicable for random mutagenesis in the sequenced *C. glutamicum *type strain ATCC 13032 because of similar endogenous insertion sequences, pronounced target site preferences, or low transposition frequencies. For instance, seven copies of IS*L3 *family-like sequences in the ATCC 13032 genome prevent the usage of IS*31831 *[18] and related elements (e.g. IS*1207*) [24] as well as their derived transposons (e.g. Tn*5531*) [24]. Very recently a mutagenesis system was described that used IS*31831*- and Tn*5*-based minitransposons to generate a comprehensive library of the *C. glutamicum *strain R which covers nearly 80% of the presently unpublished genome [57]. The *C. glutamicum *strain R has the advantage of not possessing insertion elements of the IS*31831 *family. In contrast to this, IS*6100 *is absent from the strain ATCC 13032 chromosome, ruling out integration by homologous recombination and thus it is suitable to perform transposon mutagenesis in this strain. Beside IS*6100 *quite a few active mobile elements have been published for *C. glutamicum *which are of restricted usability of generating a comprehensive random transposon library since they prefer specific sequences, e.g. a triple A or T (IS*1249*) [20] or palindromic sequences (e.g. IS*14999*) [23], as a target for integration. On the other hand, prior studies with IS*6100*-based transposons suggested independent transposition and absence of a target site preference. This was first shown in *Streptomyces lividans *and *S. coelicolor *by nucleotide sequence comparison for a small number of mutants [58] and, on the basis of Southern hybridization studies in *S. lividans *[59] and *C. glutamicum *[31].

In this study, the usability of IS*6100 *for mutagenesis in the type strain ATCC 13032 was shown by analysis of a larger number of clones. The sequence data determined from 172 insertions delivered definite position informations and, consequently, allowed comprehensive analyses on the transposon target sites. Each clone investigated carried the transposon in a different chromosomal location. The absence of regional preferences together with the absence of sequence preferences (sequence pattern, nucleotide-usage for the TSD positions or G+C content) as a target demonstrated the randomness of the library constructed and applied in auxanography analyses. Furthermore, this system might be a practicable genetic tool in other organisms as well because of the broad host-spectrum of the IS*6100 *element.

This study integrated genomic sequence data with genome-scale auxotrophy analyses. By means of auxanographic assays, for 63% of the 295 isolated auxotrophic mutants with distinct phenotypes various nutritional requirements could be identified. Additionally, out of this contingent the different integration loci of 101 clones were determined. The value of 2.9% auxotrophic clones only slightly differs from those in other transposon mutagenesis studies (1.3% [24], 2.5% [35] and 2% [36]) but is remarkably higher compared to studies in which auxotrophic mutants were obtained with frequencies of 0.2% in "*Brevibacterium flavum" *with IS*31831 *[18], 0.2% in *C. glutamicum *with IS*1249 *(Tn*5432*) [20] and 0.5% in *Streptomyces avermitilis *with IS*6100 *[56]. This might be explained by the fact that latter systems use mobile elements for which an insertion sequence specificity was identified (IS*31831 *and IS*1249*).

Transposon integrations were found in a variety of known amino acid, nucleotide and vitamin pathway genes as well in genes encoding hypothetical proteins or such of presently unknown function. The vast majority of the observed auxotrophic phenotypes could be correlated and explained with the knowledge of the mutated genomic region since most genes of amino acid, nucleotide and vitamin biosyntheses are annotated in *C. glutamicum*. In contrast, for some observed phenotypes the connection between gene and auxotrophy is not yet clear, delivering interesting targets for further studies. The loss of gene products associated with *de novo *synthesis or recycling pathways may be compensated by influx of necessary metabolite entries from other pathways that share common intermediates or precursors to a given intermediate. For instance, the PurF protein, an amidophosphoribosyltransferase, is known to be involved in more than one pathway. In *Salmonella typhimurium *PurF is the first of five enzymes shared by the *de novo *purine and HMP (hydroxymethylpyrimidine) synthesis, an essential compound of thiamine biosynthesis. Thus, PurF is expected to be required for both purine and thiamine biosynthesis [60]. It is not obvious why a *purF *transposon mutant is supplementable solely by hypoxanthine. Alternative pathways were discovered which could bypass the requirement for all *pur *genes in thiamine synthesis [61,62]. Therefore, hypoxanthine, a purine derivative, might be sufficient to compensate the growth-deficiency of this mutant.

It is known that transposon insertions near the beginning or within an operon can attenuate or interrupt the expression of downstream genes preventing RNA polymerase readthrough [63,64]. A polar effect on downstream genes was experimentally shown for an IS*6100 *integration in the gene *cysI *(*cg3118*) with transcriptional analysis using Real Time RT-PCR [65]. This example points out that the transposon system is not only applicable for high density mutagenesis of chromosomal regions (e.g. tryptophan operon, Fig. 2) but might be utilised for the determination of operons.

The screening approach for characterization of auxotrophic mutants used in this study shows a limitation in resolving complex growth phenotypes, exhibited by one thirds of these mutants. However, determination of transposon positions in randomly selected members of this auxotrophy category revealed for example mutations within *purC*, *purE*, and *purK*, genes known to be involved in purine biosynthesis. Such genes, as mentioned above, might be of interest to be investigated in further experimental studies. These findings indicate that also the complex phenotypes are not a result of additional spontaneously occurring mutations, but caused by disruption of the corresponding gene or by the accompanying polar effect.

For detailed studies on gene functions, transposon mutants have to be confirmed experimentally in order to rule out secondary mutations, polar effects or leaky phenotypes. In this study, this was carried out by a concrete example in which, by the means of supplementation, genetic deletion and complementation assays, the last gap in the histidine biosynthesis pathway of *C. glutamicum *could be closed.

Initial similarity searches revealed that the Cg0910 protein (HisN), together with another four paralogs in the *C. glutamicum *genome, apparently belongs to the monophosphatase-family proteins that usually hydrolyse the ester bond of *myo*-inositol-1(or4) phosphate. Inositol monophosphatases (IMP) play a crucial role in the biosynthesis of inositol and inositol phospholipids [66]. The role of IMP in bacteria is not completely clear yet. Bacteria of the genus *Mycobacterium *contain a number of inositol-derived cell wall constituents, like phosphatidylinositol (PI), phosphatidylinositol mannosides (PIM), lipoarabinomannan (LAM) and lipomannan (LM) [67-69]. LAM-like molecules and other inositol-containing phospholipids are not only present in mycobacteria but also in other Actinobacteria, including the genus *Corynebacterium *[70-72]. Actually, little is known about inositol synthesis in bacteria. The known *de novo *pathway in mycobacteria basically comprises the cyclization of glucose-6-phosphate to inositol-1-phosphate (I-1-P) and, subsequently, the dephosphorylation of I-1-P by IMP producing inositol [73].

Although the IMP-like proteins analysed in this study appear to be sequence homologs phylogenetic analysis revealed that they are significantly different as they branch into distinct classes. Despite their amino acid conservation, they seem to form a protein family of diverse functions and/or with diverse substrates. The assumption of different substrate specificities corresponds to previously published studies performed either in closely related mycobacteria or in *E. coli*.

The *E. coli *CysQ homolog is required for cysteine synthesis during aerobic growth. The sulfate assimilation branch of the cysteine pathway comprises sulfate uptake, its activation by formation of adenosine 5'-phosphosulfate (APS) and conversion to 3'-phosphoadenosine 5'-phosphosulfate (PAPS) by APS kinase, and its reduction to sulfite. It has been suggested that CysQ acts on PAPS as a target. It is proposed to help controlling the levels of PAPS, which may be toxic to the cell in high concentrations, or the generation of sulfite [53]. The actinobacterial CysQ IMP homologs in the phylogenetic tree branch later as compared to CysQ of *E. coli*, thus functional differences between the *E. coli *and the actinobacterial CysQ proteins might be possible. In *C. glutamicum *an APS kinase homolog is missing and thus PAPS as an intermediate is not formed. Sulfite is released from APS by direct reduction through APS reductase [65]. Therefore, the target substrate for a PAPS CysQ protein would be missing as well. It might be considered that CysQ in *C. glutamicum *and the other actinobacterial members of this phylogenetic class indeed does not possess a PAPS phosphatase activity, but most likely an inositol-phosphate phosphatase activity.

Initially, *impA *has been proposed to encode the missing HolPase, as it is clustered with the histidine biosynthesis genes in the Actinobacteridae [74]. This theory has been falsified for a *M. smegmatis impA *mutant. This mutant is not auxotrophic for histidine, but exhibits altered cell envelope permeability properties, with a notable reduction in the synthesis of phosphatidylinositol dimannoside, the precursor of LAM [54].

The SuhB inositol monophosphatase activity has been characterized biochemically in *M. tuberculosis*. Inositol-1-phosphate was shown to be the preferred target of SuhB for dephosphorylation in order to provide the PI synthase with inositol [75].

The specificity of Cg0910 (HisN) for histidinol phosphate was experimentally proven for *C. glutamicum*. For all other members of the phylogenetic IMP class labelled Cg0910 the intrinsic HolPase function could be proposed because of the close relationship and the high sequence conservation. Furthermore, in the respective Actinobacteria a protein having the enzymatic function of histidinol phosphate dephosphorylation was not identified.

The Cg0911 class comprises only corynebacterial members and one *Nocardioides *subspecies. The fact that in all these organisms *cg0911 *and *cg0910 *orthologs are located next to each other leads to the assumption of a recent gene duplication that exclusively occurred in corynebacteria or in a common ancestor. However, the finding that the respective proteins of *cg0910 *and *cg0911 *are members of different IMP classes indicate that they accomplish different functions and thus solely the *cg0910 *gene encodes the HolPase activity in *C. glutamicum*. Additional evidence that the unknown Cg0911 function, in contrast to Cg0910, does not play an important role in the cell was obtained by the inactivation of the respective gene (data not shown). The mutant showed no obvious phenotype under the applied growth conditions.

## Conclusion

In conclusion, the random transposon system described in this study together with the availability of the complete genome sequence of *C. glutamicum *ATCC 13032 [10] represents a powerful tool for genome-scale functional analyses in this type strain. Moreover, the transposon library is a resource of specific mutants which contribute to the genetic understanding of this organism in the future.

The closure of the last gap in the histidine biosynthesis pathway by identification of the *hisN *gene encoding a histidinol-phosphate phosphatase not only complements the knowledge of the pathway in *C. glutamicum *but in the entire class of Actinobacteria as well as in several Proteobacteria and Chlorobia [76]. Furthermore, with the classification of the inositol monophosphate proteins based on sequence similarities, it could be proposed that the IMP homologs in Actinobacteria, although generally acting on phosphorylated metabolites, appear to have diverse substrate specificities.

## Methods

### Bacterial strains, plasmids, oligonucleotides and culture conditions

Relevant bacterial strains, plasmids, the transposon vector and oligonucleotides constructed and used in this study are listed in Table 3. *E. coli *and *C. glutamicum *strains were routinely grown on Tryptic Soy Broth (TSB) complex media (Merck, Darmstadt, Germany) supplemented with 1.5% (w/v) agar (Invitrogen, Karlsruhe, Germany) at 37°C and 30°C, respectively. Antibiotics used for selection were kanamycin (50 μg ml^-1 ^for *E. coli *and 25 μg ml^-1 ^for *C. glutamicum*), tetracycline (5 μg ml^-1^) and nalidixic acid (50 μg ml^-1^). The oligonucleotides used as primers were designed with the Clone Manager software (Scientific & Educational Software, Cary, USA) and purchased from Operon (Cologne, Germany). The optical density of a culture in liquid medium was determined at a wavelength of 600 nm with a BioPhotometer (Eppendorf, Hamburg, Germany) spectralphotometer.

**Table 3:** Bacterial strains, plasmids and oligonucleotides used in this study

**Strain, plasmid, oligonucleotide**	**Relevant genotype/characteristics/information or sequence **^a^	**Source/reference**
***E. coli***		
DH5αMCR	F^- ^*endA1 supE44 mcrA thi-1 λ*^-^*recA1 gyrA96 relA1 deoR *Δ*(lacZYA-argF)*U169 (Φ80d*lacZ*ΔM15) Δ*(mrr-hsdRMS-mcrBC)*	[104]
		
***C. glutamicum***		
ATCC 13032	Nx^r^, wild-type	ATCC ^b^
RES167	Nx^r^, Δ(*cglIMR, cglIIR*) Restriction deficient mutant of *C. glutamicum *ATCC 13032	[79]
CG281	Δ*cg0910*	This study
		
**Transposon vector**		
pAT6100	RP4*mob*, *oriV*_E.c._, Km^r^ Mobilizable cloning vector pK18*mob2 *carrying the IS*6100 *insertion element	[31]
		
**Plasmids**		
pK18*mobsacB*	*sacB*, *lacZα*, mcs, Km^r^/mobilizable *E. coli *cloning vector, allows selection for double-crossover in *C. glutamicum*	[5]
pK18*mobsacB-Δcg0910*	pK18*mobsacB *with defined deletion derivative of *cg0910*	This study
pEC-XT99A	Tc^r^, *lacI*^q^, mcs, P_*trc*_ inducible *E. coli – C. glutamicum *shuttle expression vector	[52]
pEC-XT99A-*cg0910*ex	pEC-XT99A with internal *cg0910 *ex^c ^fragment	This study
		
**Oligonucleotides 5'-3'**		
IS6100x	GGTACAGGTAGGTCCACTTG	This study
IS6100y	CGGCAGGTGAAGTATCTCAA	This study
gs_trpP	CACCTGCATCAAGGTCGATT	This study
s6100e	GCGCCTTGTGGAGAGAGCTT	This study
s6100x	CGGATAGCGACAATACCAGC	This study
cg0910_del1	GATCTAGGATCCGATGGCACCTACAACTTCAC	This study
cg0910_del2	AACGATCCAGCGTCTCATCGTCGGCAAGTTCGGCAAGTTC	This study
cg0910_del3	CGATGAGACGCTGGATCGTT	This study
cg0910_del4	GATCTACTCGAGAGCTCTTCCAGCGTTCCATT	This study
cg0910_ex1	GATCTACAATTGAAAGGAGGACAACCATGAGCAAATATGCAGACGA	This study
cg0910_ex2	GATCTAGGATCCCTATTTTAAACGATCCAGCG	This study

^a ^r superscript indicates resistance. Nx, nalidixic acid; Km, kanamycin; Tc, tetracycline.

^b ^ATCC; American Type Culture Collection, Rockville, MD, USA.

^c ^the postfix ex indicates genes preceded by an artificial RBS

### DNA isolation, manipulation and transfer

Plasmid DNA was extracted from *E. coli *and *C. glutamicum *by an alkaline lysis technique using the QIAprep Spin Miniprep Kit (Qiagen, Hilden, Germany) with a preliminary incubation for 2 h at 37°C in resuspension buffer P1 containing 50 mg ml^-1 ^lysozyme (185,000 U mg^-1^; Serva, Heidelberg, Germany) for *C. glutamicum*. DNA modification, analyses by gel-electrophoresis and ligation were carried out by standard procedures [77]. Restriction endonucleases and T4 DNA ligase, together with enzyme buffers, were purchased from Fermentas (St. Leon-Rot, Germany) and used according to the manufacturer's instructions. DNA restriction fragments required for cloning were recovered from agarose gels by using QIAEX II gel extraction kit (Qiagen). *E. coli *and corynebacterial cells were transformed by electroporation [78] using the Bio-Rad Gene Pulser system (Bio-Rad, Munich, Germany).

### Transformation of *C. glutamicum*

The artificial transposon vector pAT6100 was electrotransferred into the restriction-deficient strain *C. glutamicum *RES167 by means of a method previously described [79]. To force transposition, the cells were additionally incubated by shaking at 200 rpm for 50 min at 37°C directly after electroporation procedure before pelleting and spreading them on solid complex media plates containing 25 μg ml^-1 ^kanamycin to select transformants. Kanamycin-resistant colonies were picked with sterile sticks, transferred to separate wells of 96-well microtiter plates (Greiner, Solingen, Germany) containing 200 μl selective complex medium and inoculated for 48 h at 30°C. Long-term storage was carried out in 96-well microtiter plates containing 10% Hogness modified freezing medium (HMFM), composed of 87% (v/v) glycerol, 1.7 mM tri-sodium citrate, 6.8 mM (NH_4_)_2_SO_4_, 0.4 mM MgSO_4 _· 4 H_2_O, 36 mM Na_2_HPO_4 _· 2 H_2_O, 13.2 mM KH_2_PO_4_, and 90% TSB at -80°C.

### Determination of transposon integration sites

Genomic DNA from *C. glutamicum *transposon mutants was extracted using the GenElute Bacterial Genomic DNA Kit (Sigma-Aldrich, Taufkirchen, Germany). Cloning and sequencing of insertion sites were carried out by plasmid rescue techniques as described before [20] with the exception that the chromosomal DNA was digested with EcoRI and XbaI to clone either side of the insertion. Sequencing of the resulting plasmids was performed with the synthetic oligonucleotide primers s6100e and s6100x for the EcoRI and the XbaI sites, respectively, by IIT Biotech (Bielefeld, Germany).

### Sequence data/accession numbers

Nucleotide homology searches were applied against the annotated *C. glutamicum *ATCC 13032 genome sequence [GenBank:BX927147] [10,80] obtained from GenBank [81]. Amino acid sequences of predicted inositol monophosphatase proteins were obtained from the Swissprot/TrEMBL databases [82]: Cg0910 [Swiss-Prot:Q8NS80], Cg0911 [Swiss-Prot:Q6M6Y2], CysQ [Swiss-Prot:Q8NS37], SuhB [Swiss-Prot:Q6M4B2] and ImpA [Swiss-Prot:Q8NNT8]. Further protein sequence information used for comparative analyses were retrieved from the following GenBank genome entries: [GenBank:AE014295] *Bifidobacterium longum *NCC2705, [GenBank:BA000035] *C. efficiens *YS-314, [GenBank:BX248353] *C. diphtheriae *NCTC13129, [GenBank:CR931997] *C. jeikeium *K411, [GenBank:U00096] *Escherichia coli *K-12, [GenBank:CP000249] *Frankia sp*. CcI3, [GenBank:AE016822] *Leifsonia xyli subsp. xyli *str. CTCB07, [GenBank:AE016958] *Mycobacterium avium subsp. paratuberculosis *K-10, [GenBank:AL450380] *M. leprae *TN, [GenBank:AL123456] *M. tuberculosis *H37Rv, [GenBank:AP006618] *Nocardia farcinica *IFM 10152, [GenBank:AE017283] *Propionibacterium acnes *KPA171202, [GenBank:BA000030] *Streptomyces avermitilis *MA-4680, [GenBank:AL645882] *S. coelicolor *A3(2), [GenBank:CP000088] *Thermobifida fusca *YX, [GenBank:AAHG00000000] *Arthrobacter sp*. FB24, [GenBank:AAGP00000000] *Brevibacterium linens *BL2, [GenBank:AAII00000000] *Frankia sp*. EAN1pec, [GenBank:AAMN00000000] *Janibacter sp*. HTCC2649, [GenBank:AAEF00000000] *Kineococcus radiotolerans *SRS30216, [GenBank:AAJB00000000] *Nocardioides sp*. JS614, and [GenBank:AAEB00000000] *Rubrobacter xylanophilus *DSM 9941.

### Bioinformatic analyses and tools for interpretation of *C. glutamicum *sequences

Database searches and sequence similarity-based searches with nucleotide and amino acid sequences were performed with the BLASTN- and BLASTP- algorithms [83], respectively. The multiple amino acid sequence alignment of the inositol monophosphatases-family proteins was performed with the DIALIGN2 software [84]. The phylogenic tree was calculated using the neighbour-joining method [85] integrated in the CLUSTALX software package [86] and visualized as a radial tree with the interactive phylogenetic tree plotting program TreeTool [87]. Sequence pattern searches were performed with the TEIRESIAS algorithm [33] provided by the IBM Bioinformatics Group.

### Auxotrophy screening and supplementation of auxotrophic phenotypes

The identification of auxotrophic mutants was performed by parallel plating on minimal media MM1 (MMYE without yeast extract [88]) and TSB complex media. The auxanographic characterizations were carried out by picking the colonies on MM1 plates supplemented with growth factors which were added as sterile filtered stock solutions by means of the purchaser's instructions, following a plating pattern described by Holliday [34], and 25 μg ml^-1 ^kanamycin. The list of growth factors comprises 20 biogenic L-amino acids (alanine, arginine, asparagine, aspartic acid, cysteine, glutamine, glutamic acid, glycine, histidine, isoleucine, leucine, lysine, methionine, phenylalanine, proline, serine, threonine, tryptophan, tyrosine, valine; final concentrations 1 mM each), nucleic acid bases (adenine, cytosine, guanine, thymine, uracil; 1 mM final cc. each), vitamins and precursors (thiamine (vitamin B1; 500 μg l^-1^), riboflavin (B2; 200 μg l^-1^), nicotinic acid (B3; 400 μg l^-1^), pantothenic acid (B5; 400 μg l^-1^), pyridoxine (B6; 400 μg l^-1^), cobalamin (B12; 10 μg l^-1^), folic acid (20 μg l^-1^), p-aminobenzoic acid (200 μg l^-1^), inositol (20 μg l^-1^), ornithine (1 mM), and hypoxanthine (1 mM)). The chemicals were purchased from VWR (Darmstadt, Germany) and Merck.

### PCR techniques and conditions

DNA fragments for deletion and complementation experiments were amplified from chromosomal DNA of *C. glutamicum *ATCC 13032 using *Pwo *DNA polymerase (Invitrogen), and *Taq *DNA polymerase (Peqlab, Erlangen, Germany) for the verification of chromosomal deletions. PCR reaction conditions were as follows: initial denaturation at 94°C for 2 min followed by 35 cycles of denaturation for 30 s, annealing for 1 min at a primer-dependent temperature, extension at 72°C for 2 min and a final extension for 4 min at 72°C. PCR products were purified using the QIAquick PCR purification kit (Qiagen).

PCR mutant screening was carried out with Eppendorf *Taq *DNA polymerase (Eppendorf) with pooled transposon mutants as templates, IS*6100*-specific primers IS6100x and IS6100y for both possible orientations of the transposon and a position-specific search primer gs_trpP (Table 3). The basic procedure was adapted from a method published by Hobom *et al*. [44]. Iterative rounds of PCR experiments were performed in order to identify the clone of interest in the library. In the first round clone-pools were examined containing all mutants in one microtiter plate. Sequential PCR rounds were carried out with the mutant-pools representing the clones of those individual plates that were tested positively in prior rounds until the exact position of the clone in the library could be determined. The bacterial cells were directly set in the PCR reaction and lysed with an initial PCR step of 94°C for 10 min to release the chromosomal DNA. PCR reaction conditions were as follows: initial denaturation at 94°C for 10 min followed by 40 cycles of denaturation for 30 s, annealing for 45 s at a primer-dependent temperature, extension at 72°C for 2 min and a final extension for 4 min at 72°C. PCR experiments were performed with the delivered buffers according to the manufacturer's instructions and with the use of a DNA Engine DYAD thermocycler from MJ Research (Watertown, MA, USA).

### Deletion and genetic complementation of *cg0910*

Defined chromosomal deletions within the *cg0910 *gene were constructed with the pK18*mobsacB *vector system which allows to identify an allelic exchange by homologous recombination [5]. The deletion was introduced into the target gene by "gene SOEing" [51]. Therefore, upstream and downstream regions of the gene to be deleted were amplified by two different PCR reactions using primer pairs cg0910_del1+2 and cg0910_del3+4 (Table 3), respectively. After purification the resulting amplificates were used as templates for the second round of PCR. The final products were digested with the restriction enzymes BamHI and XhoI, corresponding to the cleavage sites introduced via the PCR primers, and subsequently cloned into appropriately digested pK18*mobsacB *vector. The ligation mixture was used to transform *E. coli *DH5αMCR and the transformants were selected on TSB plates containing 50 μg ml^-1 ^kanamycin and 40 mg l^-1 ^X-Gal (5-bromo-4-chloro-indolyl-β-D-galactopyranoside). The resulting deletion plasmid pK18*mobsacB-Δcg0910 *was extracted and transformed into *C. glutamicum *ATCC 13032. Integration of the introduced plasmid into the chromosome by single-crossover was selected on TSB plates supplemented with 25 μg ml^-1 ^kanamycin. The kanamycin-resistant clones were grown overnight in liquid media and spread on TSB plates containing 10% (w/v) sucrose. Colonies from this plates were tested for the desired kanamycin-sensitive and sucrose-resistant phenotype by parallel picking. PCR experiments were used to verify the deletion in the *C. glutamicum *chromosome. Construction of plasmid pEC-XT99A-*cg0910*ex for *in trans-*expression of the Cg0910 protein was initiated with the amplification of the corresponding gene by PCR using the primer pair cg0910_ex1 and cg0910_ex2 (Table 3). Primer cg0910_ex1 was provided with a sequence of an optimal ribosome binding site upstream from the *cg0910 *start codon. The resulting 821-bp DNA fragment was purified and cleaved using MunI and BamHI. Appropriate restriction sites were added with the 5'-extension of the PCR primers. The fragment was subsequently ligated into the pEC-XT99A vector, which was prior cut with the corresponding enzymes. The ligation mixture was used for transformation of *E. coli *DH5αMCR, the transformants were selected on TSB plates containing 5 μg ml^-1 ^tetracycline.

## Authors' contributions

SM carried out the experimental work and drafted the manuscript. AL participated during plasmid rescue analyses. CR constructed the *cg0910 *deletion mutant and performed the phylogenetic analyses. LG performed the cointegrate stability assays. AT provided the transposon vector and participated in supervision. AP aided in coordination and participated in supervision. JK conceived of the study and participated in coordination and supervision. All authors read and approved the final manuscript.
